# The Health-Promoting Properties and Clinical Applications of Rice Bran Arabinoxylan Modified with Shiitake Mushroom Enzyme—A Narrative Review

**DOI:** 10.3390/molecules26092539

**Published:** 2021-04-27

**Authors:** Soo Liang Ooi, Sok Cheon Pak, Peter S. Micalos, Emily Schupfer, Catherine Lockley, Mi Houn Park, Sung-Joo Hwang

**Affiliations:** 1 School of Biomedical Sciences, Charles Sturt University, Bathurst, NSW 2795, Australia; sooi@csu.edu.au (S.L.O.); pmicalos@csu.edu.au (P.S.M.); eschupfer@csu.edu.au (E.S.); clockley@csu.edu.au (C.L.); 2 EROM R&D Center, EROM Co., Ltd., Chuncheon-si 24427, Gangwon-do, Korea; mhpark@eromplus.com (M.H.P.); mcity007@gmail.com (S.-J.H.); 3 Love Care Clinic, Bundang-gu, Seongnam-si 13524, Gyeonggi-do, Korea

**Keywords:** complementary therapy, BioBran, MGN-3, rice bran exo-biopolymer, functional food, nutraceuticals

## Abstract

Rice bran arabinoxylan compound (RBAC) is derived from defatted rice bran hydrolyzed with *Lentinus edodes* mycelial enzyme. It has been marketed as a functional food and a nutraceutical with health-promoting properties. Some research has demonstrated this rice bran derivative to be a potent immunomodulator, which also possesses anti-inflammatory, antioxidant, and anti-angiogenic properties. To date, research on RBAC has predominantly focused on its immunomodulatory action and application as a complementary therapy for cancer. Nonetheless, the clinical applications of RBAC can extend beyond cancer therapy. This article is a narrative review of the research on the potential benefits of RBAC for cancer and other health conditions based on the available literature. RBAC research has shown it to be useful as a complementary treatment for cancer and human immunodeficiency virus infection. It can positively modulate serum glucose, lipid and protein metabolism in diabetic patients. Additionally, RBAC has been shown to ameliorate irritable bowel syndrome and protect against liver injury caused by hepatitis or nonalcoholic fatty liver disease. It can potentially ease symptoms in chronic fatigue syndrome and prevent the common cold. RBAC is safe to consume and has no known side effects at the typical dosage of 2–3 g/day. Nevertheless, further research in both basic studies and human clinical trials are required to investigate the clinical applications, mechanisms, and effects of RBAC.

## 1. Introduction

**Figure 1:**
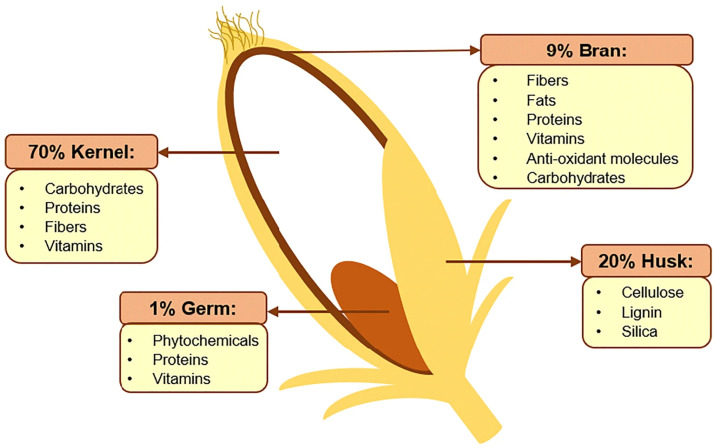
Schematic representation of rice before the milling process with the percentage of all its components and the main constituents. Reprinted under CC-BY 4.0 license from Fraterrigo Garofalo S., Tommasi T., & Fino, D. (2021). A short review of green extraction technologies for rice bran oil. *Biomass Conv. Bioref., 11*, 569–587.

Rice bran is the rough brown layer sandwiched between the outer husk of paddy and its endosperm (kernel), as shown in Figure 1. The bran constitutes roughly 8% of a rice grain before milling and is a source of many nutrients, such as lipids (20–29%), carbohydrates (20–27%, including fibers), proteins (10–15%), B-complex vitamins, minerals, as well as a variety of phytochemicals and antioxidants [1,2]. Many of the bioactive compounds within rice bran possess cancer-preventing properties, including γ-oryzanol, ferulic acid, caffeic acid, tricin, coumaric acid, phytic acid, tocopherols, phytosterols, and carotenoids [3]. As such, this by-product of rice production is now gaining increasing attention for its nutritional, functional, and commercial potentials beyond its traditional use as animal feed [4]. For instance, there is a growing use of defatted rice bran as a functional ingredient in food production, such as baking, to increase the protein, dietary fiber, and antioxidant contents [5].

A primary component of the dietary fibers found in rice bran is arabinoxylan, a hemicellulose type belonging to the non-starch polysaccharide group. The structure of arabinoxylan consists of backbone chains of β-(1-4)-linked D-xylopyranosyl (xylan) with α-L-arabinofuranose units linked as side chains via the second and third carbon positions [6]. Along with arabinose, some galactose, xylose, and glucuronic acid residues can also exist as side branches. With a molecular weight ranging between 10 to 10,000 kDa, the cross-links of many side chains make arabinoxylan mostly resistant to extraction by water [7]. Additional treatments using enzymes, alkali solutions or mechanical methods are needed to remove arabinoxylans from the stable cross-link networks to produce soluble hemicellulose and arabinoxylan compounds with low molecular weights [7,8].

One approach to facilitate the extraction of arabinoxylan from rice bran is via the use of mycelium enzymes. In one study, rice bran was treated with enzymes cultured from nine different fungi types and tested for their macrophage stimulating activities [9]. Treatment with the enzyme from *L. edodes* (shiitake mushroom) exhibited the highest macrophage stimulating activity. Further research has shown that this rice bran arabinoxylan compound (RBAC) possesses immunomodulating, anti-inflammatory, antioxidant, and anti-angiogenesis properties. From this, RBAC has shown significant potential for clinical applications as a nutraceutical [8].

This article reviews available in vivo, in vitro, and human studies from the literature to uncover the health-promoting properties of RBAC. Potential clinical applications of RBAC in cancer, human immunodeficiency viruses (HIV) infection, diabetes, irritable bowel syndrome (IBS), liver diseases, chronic fatigue syndrome (CFS), and the common cold, which are supported by research evidence, will also be presented.

## 2. The Production and Composition of RBAC

RBAC is currently available as dietary supplements or nutraceuticals for immune system improvement and disease prevention. Most prominently, a functional food called Biobran MGN-3 is developed in Japan by Daiwa Pharmaceutical Co., Ltd. This product is marketed worldwide under many different brand names, such as Biobran, Ribraxx (in Australia), Lentin Plus (in Asia), or BRM4 (in the United States) [10]. Rice bran exo-biopolymer is another variant of RBAC independently developed in Korea by Erom Co., Ltd. [8,11,12]. It is the main ingredient of Erom’s immune-related products distributed not only in Korea but also in the United States and other countries. This review adopts RBAC as the generic name for any hydrolyzed extract of defatted rice bran modified with the *L. edodes* mycelial enzyme.

**Figure 2:**
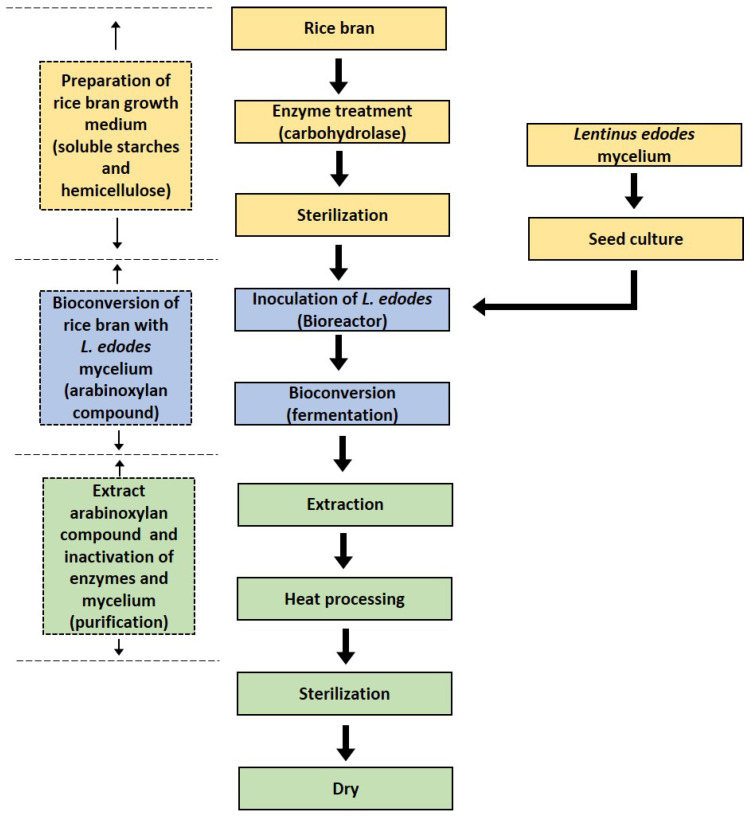
The steps to produce rice bran arabinoxylan compound: (1) Preparation: Defatted rice bran is enzymatically treated and *Lentinus edodes* is prepared as a seed culture. (2) Bioconversion: Inoculation of *L. edodes* as a bioreactor to instigate fermentation. (3) Extraction: The arabinoxylan compound is extracted through heat processing, sterilization and drying.

The production of RBAC is depicted in the following Figure 2.

Biobran Research Foundation [10] described the steps to produce RBAC as follows:

Preparation: Defatted rice bran is thoroughly mixed with hot water at the ratio of 1:5. The insoluble polysaccharide is removed from the mixture leaving only soluble starch. Glucoamylase is then added to hydrolyze the starch by cleaving the 1,4-α-glycosidic bonds of the nonreducing end of the glycosidic chains resulting in the release of d-glucose. This process increases the content of fermentable carbohydrates [13]. *L. edodes* is cultured in a liquid medium. The mycelia of *L. edodes* and any insoluble residues are then removed to obtain the carbohydrase complexes, including xylanase, glucosidase, monosidase, and hemicellulose [10].

Bioconversion: This step involves mixing and heating the hemicellulose extract (from rice bran) with the carbohydrase complexes (from *L. edodes*), resulting in the bioconversion or fermentation of the raw materials.

Extraction: The bioconversion produces partially hydrolyzed rice bran hemicellulose, which has a high arabinoxylan content. The extract is then heated, sterilized, condensed, and added with excipient resulting in the final product as a powder [10].

**Figure 3:**
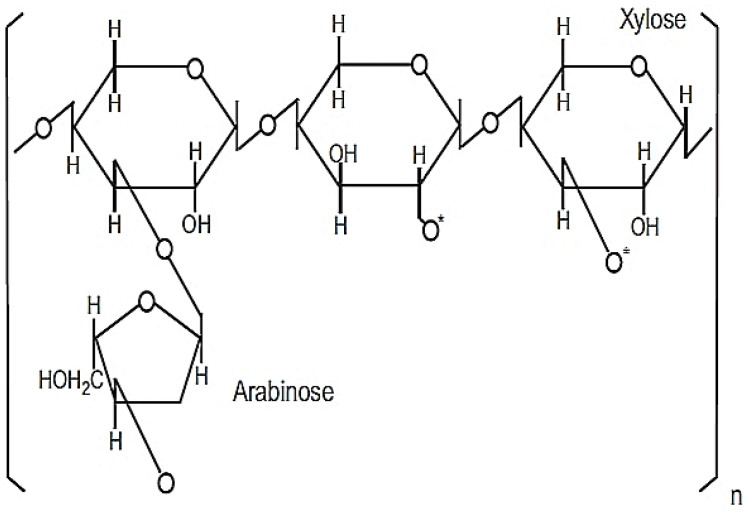
Chemical structure of MGN-3/Biobran. Reprinted from Wheat and Rice in Disease Prevention and Health, 1st ed., M. H. Ghoneum, Chapter 31—Apoptosis and arabinoxylan rice bran, pp. 401–408, Copyright 2014, with permission from Elsevier.

This powdered product is 98.4% soluble in purified water consisting of heteropolysaccharide structures with molecular weights between 30–100 kDa [14,15]. The active ingredient responsible for its immunomodulatory property is likely to be a modified arabinoxylan with a xylose in its main chain and an arabinose polymer side chain [14], as shown in Figure 3.

## 3. Health-Promoting Properties

### 3.1. Immunomodulatory Action

RBAC has been promoted as a plant-based biological response modifier that acts to modify the body’s immune response [16]. RBAC can enhance both innate and adaptive immune systems in eliminating pathogens and malignant cells.

The innate immunity is enhanced via up-regulating the cytotoxic activity of natural killer (NK) cells with increased granularity [17]. RBAC’s effects on NK cell activity were demonstrated in vivo in different mouse models [18,19,20,21,22], in vitro with different cell lines [11,19,23], and in human trials [17,23,24,25,26,27,28,29]. RBAC can also enhance the phagocytic cellular functions of macrophages, neutrophils, and monocytes. Macrophages cultured with RBAC demonstrated better efficacy in phagocytosis, enhanced spreading ability, and increased production of cytokines (tumor necrosis factor-alpha [TNF-α] and interleukin [IL]-6) to regulate B-cell function [9,12,30,31]. Similarly, neutrophils and monocytes treated with RBAC have also shown enhanced anti-bacterial activity characterized by increased oxidative burst and cytokine production in the presence of bacteria [32].

RBAC influences the adaptive immune response via the dendritic cells (DCs). Immature DCs derived from peripheral monocytes showed increased expression of maturation markers after treatment with RBAC [33,34] The effect of RBAC as a potent inducer of DC maturation and activation has also been confirmed in a placebo randomized control trial (RCT) with 48 multiple myeloma patients [28,29]. Activation of DCs also triggers T lymphocyte responses with the increased proliferation of T and B lymphocytes, as revealed in several studies [17,23,33,35,36]. In patients with underactive immune systems, RBAC may also enhance immune function by inhibiting the immunosuppression effects of Treg lymphocytes [36].

### 3.2. Anti-Inflammatory Effect

The mechanism of how RBAC can trigger the immune-inflammatory response remains unclear. Experiments by Endo and Kanbayashi [37] have confirmed that molecules of immunoreactive polysaccharides can be detected in blood after oral administration of RBAC. Due to the similarity in structure and molecular weight with lipopolysaccharide from Gram-negative bacteria, it has been suggested that enzyme-treated arabinoxylan may mimic pathogen-associated molecular patterns [6]. As lipopolysaccharide binds to the toll-like receptor 4 on phagocytes’ surface, low-molecular-weight arabinoxylan may also activate phagocytes through the same receptor [6]. RBAC administration was shown to exert transient activation of inflammatory cytokines in healthy individuals [25]. However, in the presence of infection or inflammation, it could down-regulate the overall inflammatory response through competing with pathogens at the immune-stimulating ligands [6]. Many pre-clinical [20,22,24,31,32,33,35] and clinical studies [25,28,38] demonstrated RBAC affecting the production of both pro-inflammatory (tumor necrosis factor [TNF]-α, interferon [INF]-γ, IL-1β, IL-2, IL-6, IL-7, and IL-12) and anti-inflammatory (IL-1RA and IL-10) cytokines. Hence, RBAC plays a role in regulating the immuno-inflammatory mechanism by modulating these signaling proteins in a complex multifactorial manner.

The dysfunction of localized inflammatory regulation can lead to chronic and systemic inflammation, which is the underlying cause of many chronic conditions, including asthma, autoimmune conditions, cardiovascular diseases, obesity, diabetes, depression, osteoarthritis, and cancer [39]. Ichihashi [40] reported the potential beneficial effect of RBAC on chronic inflammation in a case series. Eight patients with chronic rheumatism on steroids were given RBAC as a food supplement for 6 to 12 months. Three of them responded to RBAC, showing a reduction in rheumatoid factor and C-reactive protein (CRP) post-treatment. Ghoneum and El Sayed [41] further demonstrated that RBAC has a protective effect against neuroinflammation in a mouse model of sporadic Alzheimer’s disease by lowering IL-6 and intercellular adhesion molecule-1 in brain tissues.

RBAC was also shown in clinical trials to exert effects on lowering the biomarkers for systemic inflammation. A small study (n = 10) that combined RBAC and curcumin reported a potential effect in reducing the raising erythrocyte sedimentation rate in 44% of the participants with early B-cell lymphoid malignancies [42]. In another study involving patients with mixed-type IBS, supplementation of four weeks with RBAC was shown to have an anti-inflammatory effect by lowering CRP value and the neutrophil-to-lymphocyte ratio [43].

### 3.3. Antioxidant Action

RBAC has been shown to augment the function of the antioxidant defense system. Solutions of RBAC in ethanol were assessed by Tazawa et al. [44] as having a high scavenging rate on hypoxanthine-xanthine oxidase generated superoxide anion radicals, ferrous sulphate-hydrogen peroxide and ultra-violet light reaction system-generated hydroxyl radicals. In an animal study with Ehrlich carcinoma-bearing mice, Noaman et al. [45] reported that RBAC efficiently suppressed tumor growth with an associated normalization of lipid peroxidation and glutathione contents.

RBAC was also shown to enhance the activity of the endogenous antioxidant scavenging enzymes, which include superoxide dismutase (SOD), glutathione peroxidase (GPx), catalase (CAT) and glutathione-S-transferase (GST), in blood, liver, and tumor tissue [45]. Similarly, the messenger ribonucleic acid (mRNA) expressions of GPx, SOD1 and CAT in the liver were up-regulated, demonstrating RBAC’s protective properties against oxidative stress [45].

In another murine model, sporadic Alzheimer’s disease was induced via intracerebroventricular injection of streptozotocin [41]. After administering RBAC for 21 days, the streptozotocin-treated mouse showed improvement in oxidative stress biomarkers with the significant increase of malondialdehyde and glutathione levels in the hippocampus (*p* < 0.0001). Oxidative stress was further studied via the expression of nuclear factor erythroid 2-related factor 2 (Nrf2) and antioxidant response element (ARE). RBAC was shown to increase the hippocampal Nrf2 and ARE levels significantly (*p* < 0.0001) in a dose-dependent manner where a high level of RBAC (200 mg/kg) approximately returned the Nrf2 and ARE levels to that of control [41].

This protective effect against oxidative stress was also shown in another murine study examining gamma radiation’s effects [46]. Exposure to ionizing radiation is known to induce oxidative stress that can damage cellular macromolecules leading to the demise of hematopoietic tissues [47]. Mice exposed to gamma radiation showed significant depression in their full blood count, hypocellularity of their bone marrow, and a remarkable decrease in splenic weight. In contrast, pre-treatment with RBAC resulted in protection against such irradiation-induced damages [46]. RBAC’s ability to augment the antioxidant defense system to counteract the severe adverse effects and toxicity of radiation therapy is confirmed in a double-blind, placebo RCT with head-and-neck cancer patients (n = 65) undergoing radiotherapy by Tan and Flores [48].

### 3.4. Angiogenesis Inhibition

**Figure 4:**
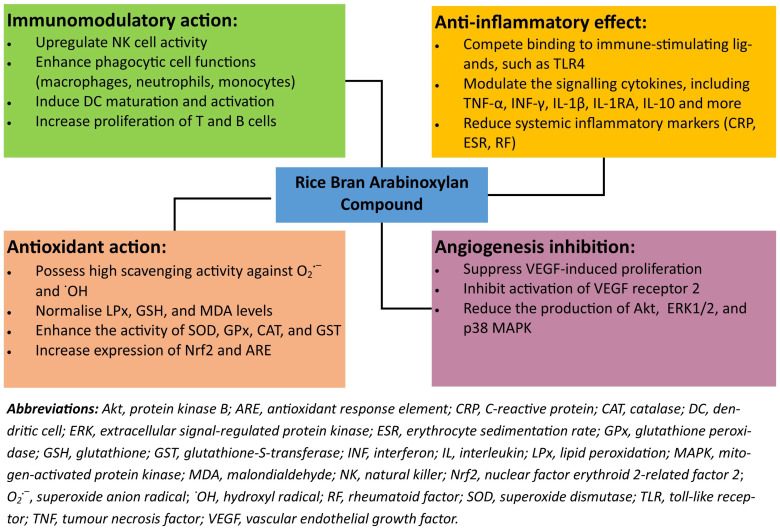
A summary of the key health-promoting attributes of rice bran arabinoxylan compound.

RBAC can affect new blood vessel growth through the vascular endothelial growth factor (VEGF) pathway. An in vitro study by Zhu et al. [49] demonstrated that RBAC significantly inhibited VEGF-induced tube formation in human umbilical vein endothelial cells co-cultured with human dermal fibroblasts. Furthermore, RBAC also suppressed the VEGF-induced proliferation and migration of human umbilical vein endothelial cells in a dose-dependent manner [49]. The anti-angiogenic mechanism of RBAC operates through the reduction of not only the VEGF-induced activation of VEGF receptor 2, but also the downstream proteins of protein kinase B, extracellular signal-regulated protein kinase 1/2, and p38 mitogen-activated protein kinase [49]. Therefore, RBAC can potentially mediate angiogenesis, implicated in the pathological progression of conditions such as cancer, cardiac and limb ischemia, diabetic retinopathy, rheumatoid arthritis, and neoplasms [50]. Figure 4 summarizes the medicinal actions and effects of rice bran arabinoxylan compound discussed in this section.

## 4. Clinical Applications

### 4.1. Cancer

The immunomodulatory effects of RBAC make it a potential anti-cancer lead compound [16]. The antiproliferative actions on cancer cells were demonstrated in several in vitro studies that cultured human and murine cancer cell lines with RBAC [19,51,52,53]. In models of animals implanted with malignancies, including gastric cancer [21], neuroblastoma [19], melanoma [11], and Ehrlich carcinoma [20,45], RBAC demonstrated its ability to inhibit cancer growth. RBAC was also shown to work synergistically with other natural anti-cancer substances, such as Baker’s yeast [51] and curcumin [53]. It also enhanced the effectiveness of chemotherapeutic agents, including cisplatin [37,54], daunorubicin [54,55] and paclitaxel [56,57] as well as radiotherapy [48,58].

Many successful RBAC-treated clinical cases have been reported in current literature with primary cancers, including leukemia, prostate, breast, colorectal, pancreatic, liver, lung, skin, and ovarian [59]. Favorable outcomes reported in these cases include improvements in tumor markers, immunocompetence profile, and initial symptoms after taking RBAC [60,61]. Some patients’ conditions stabilized and showed no indication of recurrence at follow-up [17]. Improvement in cancer patient’s quality of life (QoL) in terms of subjective sleep quality, appetite, digestion, physical activity and decrease in anxiety and pain, as well as reduced adverse events during chemotherapy, were also reported [62,63]. One case reported remission of metastatic lung tumor after 34 months of self-treatment with RBAC [64]. Complete or near-complete remission of hepatic metastases was also reported by Hajto and Kirsch [65] in seven cases of patients treated with a combination of RBAC, mistletoe lectins and wheat germ extract. In three case reports, patients with terminal cancer exceeded their initially clinically projected lifespans and reported significantly improved QoL [60,66,67].

A recent systematic review conducted by the lead authors found evidence supporting RBAC as a complementary therapy alongside conventional cancer treatment [59]. The review identified 11 RBAC clinical trials with cancer patients, including six RCTs. As an adjunct therapy during conventional chemo- and radiotherapy, the reported effects of RBAC include improvement of the immune profile, reduction of side effects, and improvement of treatment outcomes [29,68,69,70,71]. Investigating the efficacy of administering RBAC as a follow-up therapy after conventional treatment, three clinical studies [17,27,36] reported favorable results in the restoration of the immune system profiles, improvement in QoL, and enhancing the long-term survival rate of late-stage cancer patients. The review also found RBAC as safe with no related adverse events reported in the clinical trials and recommended RBAC as an adjunct or follow-up therapy to complement conventional cancer treatment [59]. A more recent trial by Tan and Flores [48] further confirmed the benefits of RBAC for head-and-neck cancer patients during radiation treatment. Patients who took RBAC before, during and after treatment had better clinical outcomes compared to the placebo group. A new study is currently underway to confirm the effects of RBAC on patients’ QoL during active cancer treatment [72]. Table 1 shows a summary of the systematic review and recent clinical trials evaluating the effects of RBAC on cancer patients.

### 4.2. HIV Infection

The anti-HIV activity of RBAC was first reported by Ghoneum [73]. In an in vitro study, the peripheral blood mononuclear cells from three healthy donors were incubated with HIV-1 SF strains (HIV-1 p24 of 3000 pg/106 cells) at 37 °C for one hour. The infected cells were then kept in complete media for seven days at 37 °C with or without RBAC at a range of concentrations (0, 12.5, 25, 50, 100 µg/mL). The mean production of HIV-1 p24 antigen, compared to control, was reduced by 18.3%, 42.8%, 59% and 75%, at concentrations of 12.5, 25, 50, and 100 µg/mL of RBAC, respectively [73]. Thus, RBAC inhibited HIV-1 replication in a dose-dependent manner. RBAC was also shown to inhibit HIV-induced syncytia formation. A syncytium is a fusion of an infected cell with neighboring cells forming a multinucleated product. Syncytia formation is known to contribute to virus dissemination [74]. Ghoneum investigated syncytia formation with the mononuclear cell fusion array from five patients with acquired immunodeficiency syndrome (AIDS) [73]. The mononuclear cells were cultured for seven days at 37 °C with or without RBAC at different concentrations (0, 12.5, 25, 50, 100 µg/mL). RBAC was shown to significantly inhibit the syncytia formation in a dose-dependent manner, with the maximum inhibition of 75% observed at a concentration of 100 µg/mL [73].

The effects of RBAC on the immune, hepatic, and renal functions of HIV-positive individuals were examined by Lewis et al. [75]. in a double-blind, placebo RCT. Forty-seven HIV+ participants on stable antiretroviral therapy (ART) were randomly assigned to consume either 3 g/day of RBAC (n = 22) or placebo (n = 25) supplement for six months. Participants were assessed at the third and sixth month for cluster differentiation (CD)4+ T cells and CD8+ T cells, liver enzymes, and kidney function for comparison with baseline. Both groups’ liver and kidney markers remained within normal limits throughout the study, with no significant difference between groups. However, there was a statistically significant difference in the percentage change in CD8+ T cells between the groups (F[1,32] = 4.8, *p* = 0.04), with the placebo group showing rising CD8+. Furthermore, the RBAC group showed a clinically significant increase in the CD4+/CD8+ ratio (+8.6%) compared to a decrease (−12.2%) in the placebo group, even though the change was only statistically marginal (F[1,31] = 3.2, *p* = 0.085). Elevated CD8+ T cell count under ART is recognized as an early warning indicator for future treatment failure [76]. With its ability to attenuate the CD8+ overstimulation, RBAC can potentially prevent the resultant complications of ART in HIV+ patients [75].

Another double-blind, placebo RCT for HIV+ patients using RBAC was conducted by Cadden et al. [77]. The study aimed to investigate RBAC’s anti-inflammatory effects in ART-suppressed HIV infection for its potential use in preventing end-organ disease in AIDS patients. Analysis of the biomarkers from 24 participants (RBAC = 12, placebo = 12) consisting of hsCRP, CD14, sCD163, lipoprotein binding protein, and IL-6 did not reveal any statistically significant difference between the RBAC and placebo groups after 12 weeks of supplementation. Hence, the clinical application of RBAC for the HIV+ population requires further investigation.

### 4.3. Common Cold

The common cold is an acute self-limiting viral infection of the upper respiratory tract. The preventive effect of RBAC against common cold in older adults was examined in a double-blind, cross-over RCT [78]. The study recruited 50 elderly aged 70 to 95 years from a residential care facility. Each participant consumed RBAC in one period and water-soluble fractions of rice bran as a control in another period. Each administration period was six weeks with a two-week washout interval. Participants were randomly assigned to receive either RBAC or control substance as their starting treatment in the first period. Common cold symptoms were assessed and scored by care personnel daily. Data from 36 participants who completed both periods were analyzed. Withdrawal reasons were due to healthy participants leaving the care facility and were unrelated to the treatment protocols. The control group’s total symptom score was significantly higher (*p* < 0.05), three times more than the RBAC group. Furthermore, the mean duration of common cold symptoms was shorter in the RBAC group (1.2 days) than the control (2.6 days), albeit not statistically significant. The results demonstrated that RBAC could prevent the upper respiratory tract viral infection and reduce common cold symptoms in older adults [78].

### 4.4. Liver Diseases

RBAC can potentially protect against liver injury caused by viral hepatitis. In an in vivo study by Zheng et al. [79], male Wistar rats were injected with D-galactosamine to induce liver injury similar to human hepatitis B. Rats were pre-treated with RBAC at 20 mg/kg body weight or 60 mg/kg body weight, intraperitoneally. Compared to control, pre-treated rats had significantly (*p* < 0.05) lower serum transaminase levels. The study also found that the low-molecular-weight (≤0.4 kDa) fractions of RBAC were more effective than both medium (0.4–2000 kDa) and high (>2000 kDa) molecular-weight fractions in lowering serum transaminase levels. The hepatoprotective mechanisms were partly mediated by the down-regulation of both the IL-18 mRNA expression in the liver and serum IL-18 concentration in RBAC-administered rats [79]. Further experiments also show that the suppressive effects of RBAC on hepatitis induced by D-galactosamine are related to the inhibition of nuclear factor-κB, mitogen-activated protein kinase, and CD14 expression [80].

In a non-inferiority clinical trial, two groups of hepatitis C patients were randomized to receive either RBAC (n = 16, 1 g/day) or the standard treatment (pegylated interferon plus ribavirin, n = 21) for three months [81]. Post-treatment viral load in both groups reduced similarly and significantly relative to baseline (*p* < 0.05). Treatment with RBAC was shown to significantly increase the level of IFN-γ after three months (*p* < 0.001). The standard treatment group reported adverse events, including fever, anemia, thrombocytopenia, and fatigue, whereas no adverse effects were reported in the RBAC group. Hence, RBAC could be a viable alternative to pegylated interferon plus ribavirin treatment for hepatitis C with fewer side effects [81].

RBAC supplementation also benefits patients with nonalcoholic fatty liver disease (NAFLD). In a 90-day double-blind, placebo RCT [82], 23 NAFLD patients were randomly assigned to consume 1 g/day of either RBAC or placebo. Serum biomarkers were assessed at baseline, 45, and 90 days. Alkaline phosphatase, a hallmark of liver damage, significantly decreased (*p* = 0.03) in the RBAC group compared to placebo. Significant increases in the eosinophils (*p* = 0.02), monocytes (*p* < 0.001), and IL-18 (*p* = 0.03) in the RBAC group compared to placebo were also detected at the 90-day follow-up, suggestive of the immunomodulatory effects of RBAC [82]. Decreases in both neutrophils and neutrophil-to-lymphocyte ratio in RBAC group compared to placebo were also observed, even though not statistically significant, may be clinically relevant. No adverse events caused by RBAC was reported in this study [82]. Overall, the evidence on the beneficial effects of RBAC for managing NAFLD is promising.

### 4.5. Chronic Fatigue Syndrome

The potential beneficial effects of RBAC on CFS was first reported in a small observational study with patients diagnosed with CFS (n = 10) consuming 3 g/day of RBAC for two months [83]. Symptom severity was assessed with Chalder fatigue score and the visual analogue scale at baseline and after two months. This study observed symptom improvement in patients with clear viral etiology (4 out of 10 patients). The author postulated a potential link between the increased NK cell activity during RBAC supplementation and fatigue symptoms [83].

To validate the effectiveness of RBAC in reducing fatigue in CFS patients, McDermott et al. [84] conducted a placebo RCT with 71 participants. Participants were given oral RBAC supplement (n = 37) or matching placebo (n = 34) for eight weeks at a dosage of 6 g/day (2 g × 3 times daily). The primary outcome measure was the Chalder physical fatigue score. Additional outcome measures included self-reported fatigue, self-assessment of improvement, change in key symptoms, QoL, anxiety and depression. Both groups of participants experienced marked improvement over the study duration but without significant differences between groups. As such, the study did not find RBAC to be superior to placebo for reducing fatigue in CFS patients [84]. However, this study did not differentiate patients based on their CFS aetiology, a potential factor influencing the treatment efficacy of RBAC observed by Kenyon [83].

RBAC was combined with oncothermia therapy to treat patients with CFS due to cancer treatment in an RCT [71]. Fifty patients completed the study, with 25 of them taking RBAC (1 g × 3 per day) plus receiving 15 weekly oncothermia treatment, in addition to their standard cancer treatment (chemotherapy or radiotherapy) over six months. Another 25 control patients received only standard cancer treatment. The severity of CFS was assessed with Chalder fatigue score, patient global impression of change, and QoL questionnaire (EORTC QLQ-C30). The study found a significant reduction in the mean fatigue score in the RBAC+ oncothermia group after treatment compared to baseline (*p* < 0.01), whereas no significant change in fatigue score was detected in the control group. In terms of the mean patient global impression of change, the RBAC+ oncothermia group indicated ‘much improved‘ after treatment compared to ‘no change‘ in the control group. Therefore, the study found the combined therapy (RBAC+ oncothermia) effectively reduced fatigue in cancer patients suffering from CFS [71].

Overall, the effects of RBAC on CFS remain unclear, with mixed results from the low number of studies conducted. Further research is needed to investigate the applicability of RBAC for CFS patients with the disease aetiology being a potential determinant factor for fatigue reduction.

### 4.6. Irritable Bowel Syndrome

In a pilot study by Kamiya et al. [43], 40 patients with diarrhea-predominant or mixed-type IBS were randomly assigned to receive an oral supplement of either 1g of RBAC or placebo powder twice a day for four weeks. Global symptom assessments were conducted at baseline and weekly intervals using a five-point Likert scale. Other outcome measures, including gastrointestinal-specific QoL and anxiety, were evaluated using self-reported questionnaires before and at the end of supplementation. The participants also had blood tests at baseline and week four [43].

The results showed that RBAC supplementation was effective for 63.2% of the patients RBAC group compared to only 30% in the placebo group (*p* < 0.05) at the end of the study [43]. Significant between-group differences in serum neutrophil (*p* = 0.0184) and lymphocyte (*p* = 0.0384) were also reported. A lower neutrophil-to-lymphocyte ratio in the RBAC group suggests reduced subclinical inflammation. The RBAC group also reported significant improvement in the symptoms of reflux (*p* = 0.013), diarrhea (*p* < 0.001), and constipation (*p* < 0.024) compared to baseline. No significant changes in the placebo group’s symptom ratings were detected at the end of the study, compared to baseline. However, comparisons of all symptom changes between-group were not significant due to the small sample size [43]. A full-scale trial validating the effects of RBAC on mixed-type IBS is warranted.

### 4.7. Diabetes

Ohara et al. [85,86] explored the effects of RBAC in streptozotocin-induced diabetic rats. In one study [85], the diabetic rats were administered with 0.5 g/kg body mass of RBAC via stomach tube for two months. Compared to controlled diabetic rats administered with inert substance (0.5% sodium alginate), RBAC-fed rats showed significantly reduced water intake (*p* ≤ 0.05), suggested as an improvement in polyuria. The RBAC-fed rats also had significantly (*p* ≤ 0.05) lower triglycerides, total cholesterol, total protein, and zinc levels in serum at the end of the study. However, no significant difference in body mass, serum glucose and insulin were detected between the two groups [85].

In another study [86], adult rats with diabetes induced with streptozotocin as neonates were fed with experimental diets consisting of only 1.7% cellulose or 1.7% cellulose plus 1% RBAC for 60 days. Oral glucose tolerance tests were performed after 20 h of fasting on day 58. Diabetic rats fed with RBAC, compared to control, had significantly lower glucose level (*p* < 0.05) at 30 min after being fed with glucose (2 g/kg body mass) via stomach tubes. Like the previous study, serum biochemistry analysis also revealed a significantly lower total cholesterol level (*p* < 0.05) in RBAC-fed diabetic rats than those on the control diet [86]. Supplementation with RBAC potentially improves blood glucose spike as well as lipid and protein metabolism in diabetic rats. To date, there is no published human study on the potential beneficial effects of RBAC for diabetes.

Table 2 is a summary of all clinical trials evaluating the effects of RBAC on health conditions other than cancer.

## 5. Safety and Adverse Events

The safety of RBAC has been well established in both human and animal studies. The resultant product’s median lethal dose (LD50) is above 36 g/kg, and the No-Observed-Adverse-Effect-Level is 200 mg/kg/day or above [87]. The compound also tested negative in a reverse mutagenicity array and low antigenicity in a controlled test with guinea pigs with no positive allergic responses observed [87]. Twenty-four healthy volunteers were given RBAC at different concentrations of 15, 30, and 45 mg/kg body weight daily. Blood chemistry analysis using Panel 20, which includes liver enzymes, was conducted to assess toxicity. After one month of treatment, no abnormalities were detected in these parameters, as compared to baseline [16]. RBAC also has an excellent safety record. In the systematic review of RBAC conducted by the authors summarized earlier, no adverse events at the typical dosage of 2–3 g/day, was reported in any of the included clinical trials (total 11) or clinical case reports (total 14) [59]. Hence, RBAC is considered safe to consume with no known side-effect at the typical dosages used in research and clinical settings.

## 6. Conclusions

Research evidence supports the use of hydrolyzed rice bran enzymatically modified with shiitake mushroom as a functional food and nutraceutical with health-promoting properties. RBAC is a plant-based immunomodulator that can affect both innate and adaptive responses. It balances the inflammatory cytokines, protects against oxidative stress, and inhibits the uncontrolled proliferation of new blood vessels. RBAC has been successfully applied to restore the immune profiles, improve QoL, and enhance cancer patients’ long-term survival. The available research literature also suggests that RBAC supplement can be beneficial for HIV infection, diabetes, IBS, hepatitis, NAFLD, CFS, and the common cold. Therefore, the potential clinical applications of RBAC are wide-ranging.

However, the understanding of the health-promoting properties and clinical applications of RBAC is still limited. The bulk of the available basic research was on the compound’s immunomodulatory properties, with only a small number of studies on its anti-inflammatory and antioxidant properties. More research is needed to explore the physiological pathways of RBAC exerting these effects. The anti-angiogenic property of RBAC is a new discovery from a recent study and the findings need validation.

Although there is good evidence supporting the use of RBAC as a complementary therapy in cancer, evidence for RBAC beyond cancer remains insufficient. Further research in both basic and human clinical studies are needed to establish the potential mechanisms by which RBAC may affect the pathophysiology of different disease conditions.

## Figures and Tables

**Table 1:** Summary of systematic reviews and recent clinical trials evaluating the effects of RBAC on cancer patients.

Study	Design	Condition	Objectives	N	Dosage	Findings
Ooi et al. (2018), Australia	Systematic review (includes studies until May 2017)	Various malignancies	To comprehensively review the available evidence on the effects and efficacies of MGN-3 as a complementary therapy for conventional cancer treatment.	11 studies (NRT = 5, RCT = 6). N = 566 (RBAC = 351, control = 215)	1–3 g/day with duration ranging from 2 weeks to 4 years.	Available evidence suggests RBAC as an effective immunomodulator that can complement conventional cancer treatment. More well-designed RCT is needed to strengthen the evidence base.
Tan & Flores (2020), Philippines	Double-blind placebo RCT	Head-and-neck carcinoma	To investigate the effects of RBAC on hematologic profile, nutritional status, and QoL of head-and-neck cancer patients undergoing radiation therapy or concurrent chemotherapy.	N = 65 (RBAC = 33, placebo = 32)	3 g/day. 2 weeks before the start of treatment, during treatment and 2 months after treatment.	The results showed better clinical outcomes for the RBAC group with fewer blood transfusions, treatment delays and hospital admissions, avoidance of treatment mortalities and morbidities, and improved QoL.
Ooi et al. (2020), Australia	Double-blind placebo RCT	Solid organ cancers stage II and above	To evaluate the effects of RBAC on cancer patients’ QoL, inflammatory and nutritional status, cytokine profile, and gut microbiota during active treatment, compared to placebo.	N = 50 (RBAC = 25, placebo = 25)	3 g/day for 6 months during active treatment.	This trial is ongoing. Trial Reg No: ACTRN126190005 62178p. Targeted completion date: May 2022.

Abbreviation: NRT, non-randomized trial; QoL, quality of life; RBAC, rice bran arabinoxylan compound; RCT, randomized control trial.

**Table 2:** Summary of clinical trials evaluating the effects of RBAC on health conditions other than cancer.

Study	Design	Condition	Objectives	N	Dosage	Findings
Kenyon et al. (2001), UK	Single-arm prospective study	CFS	To assess the effect of RBAC on the fatigue symptoms in patients with CFS.	N = 10	3 g/day for 2 months	In those patients with a clear viral aetiology of CFS, RBAC produced significant improvement.
Maeda et al. (2004), Japan	Double-blind cross-over, active-control RCT	Common cold	To examine the preventive effect of RBAC against the common cold symptoms in older adults (age 70–95) compared to a water-soluble rice bran supplement.	N = 36	0.5 g/day for 6 weeks	The rice bran group’s total symptom score was three times higher than that for the RBAC group. The average duration of symptoms was 2.6 days for the rice bran group, whereas it was only 1.2 days for the RBAC group.
McDermott et al. (2006), UK	Double-blind placebo RCT	CFS	To evaluate the effectiveness of RBAC as a putative natural killer cell stimulant in reducing fatigue in CFS patients.	N = 67 (RBAC = 35, placebo = 32)	2 g/day for 8 weeks	Both groups showed marked improvement over the study duration but without significant differences. The findings do not support a specific therapeutic effect for RBAC in CFS.
Kamiya et al. (2014), Japan	Double-blind placebo RCT	IBS	To investigate the immune modulation effect of RBAC in patients with diarrhea-predominant IBS or mixed IBS.	N = 39 (RBAC = 19, placebo = 20)	2 g/day for 4 weeks	RBAC group showed a significant decrease (*p* < 0.05) in the score of reflux, diarrhea, and constipation. The placebo group showed no significant difference in symptom scores.
Petrovics et al. (2016), Hungary	Double-blind, active-control RCT	CFS	To evaluate the efficacy of a combined oncothermia and RBAC therapy to treat cancer patients suffering from CFS.	N = 50 (RBAC = 25, control = 25)	1 g/day for 24 weeks	The mean fatigue score was significantly reduced (*p* < 0.01) after treatment in the intervention group but not in the control group.
Salama et al. (2016), Egypt	Double-blind RCT	HCV	To examine the anti-HCV effects of RBAC in restricting viremia in chronic HCV patients compared to the standard PEG IFN therapy.	N = 37 (RBAC = 16, control = 21)	1 g/day for 3 months	RBAC showed a similar effect in reducing HCV load compared to PEG IFN without any side effects.
Lewis et al. (2018), USA	Double-blind placebo RCT	NAFLD	To evaluate the effect of RBAC on biomarkers in adults with NAFLD.	N = 23 (RBAC = 12, placebo = 11)	1 g/day for 90 days	RBAC had beneficial effects on several biomarkers (monocytes, eosinophils, IFN-γ. IL-18), demonstrating immunomodulatory activities in patients with NAFLD.
Lewis et al. (2020), USA	Double-blind placebo RCT	HIV	To evaluate the effects of RBAC on immune, hepatic, and renal function in HIV+ individuals on stable antiretroviral therapy.	N = 47 (RBAC = 22, placebo = 25)	3 g/day for 6 months	The results showed promising immunomodulatory and anti-senescent activities of RBAC with a statistically significant decrease in CD8+ count and a clinically significant increase in CD4+/CD8+ ratio.
Cadden et al. (2020), USA	Double-blind placebo RCT	HIV	To evaluate the anti-inflammatory effects of RBAC in virologically suppressed HIV patients who had incomplete immune reconstitution.	N = 24 (RBAC = 12, placebo = 12)	3 g/day for 12 weeks	The study found no evidence of a beneficial effect of 12 weeks of RBAC supplementation compared to placebo.

Abbreviation: CD, cluster differentiation; CFS, chronic fatigue syndrome; HCV, hepatitis C virus; HIV, human immunodeficiency virus; IBS, irritable bowel syndrome; IFN, interferon; IL, interleukin; NAFLD, nonalcoholic fatty liver disease; PEG IFN, pegylated interferon; RBAC, rice bran arabinoxylan compound; RCT, randomized control trial; UK, United Kingdom; USA, United States of America.

## Data Availability

Not applicable.

## Abbreviations

The following abbreviations are used in this manuscript:

AIDS	Acquired immunodeficiency syndrome
ARE	Antioxidant response element
ART	Antiretroviral therapy
CAT	Catalase
CD	Cluster differentiation
CFS	Chronic fatigue syndrome
CRP	C-reactive protein
DC	Dendritic cells
GPx	Glutathione peroxidase
GST	Glutathione-S-transferase
HIV	Human immunodeficiency viruses
IBS	Irritable bowel syndrome
IL	Interleukin
INF	Interferon
mRNA	Messenger ribonucleic acid
NAFLD	Nonalcoholic fatty liver disease
Nrf2	nuclear factor erythroid 2-related factor 2
QoL	Quality of life
RBAC	Rice bran arabinoxylan compound
RCT	Randomized control trial
SOD	Superoxide dismutase
TNF	Tumor necrosis factor
VEGF	Vascular endothelial growth factor
